# Mucoadhesive Nanoparticles May Disrupt the Protective Human Mucus Barrier by Altering Its Microstructure

**DOI:** 10.1371/journal.pone.0021547

**Published:** 2011-06-29

**Authors:** Ying-Ying Wang, Samuel K. Lai, Conan So, Craig Schneider, Richard Cone, Justin Hanes

**Affiliations:** 1 Department of Biomedical Engineering, Johns Hopkins University School of Medicine, Baltimore, Maryland, United States of America; 2 Department of Chemical and Biomolecular Engineering, Johns Hopkins University, Baltimore, Maryland, United States of America; 3 Department of Biophysics, Johns Hopkins University, Baltimore, Maryland, United States of America; 4 Department of Ophthalmology, The Wilmer Eye Institute, Johns Hopkins University School of Medicine, Baltimore, Maryland, United States of America; 5 Department of Environmental Health Sciences, Johns Hopkins School of Public Health, Baltimore, Maryland, United States of America; 6 Center for Cancer Nanotechnology Excellence, Institute for NanoBioTechnology, Johns Hopkins University, Baltimore, Maryland, United States of America; 7 Center for Nanomedicine, Johns Hopkins University School of Medicine, Baltimore, Maryland, United States of America; University of California, Merced, United States of America

## Abstract

Mucus secretions typically protect exposed surfaces of the eyes and respiratory, gastrointestinal and female reproductive tracts from foreign entities, including pathogens and environmental ultrafine particles. We hypothesized that excess exposure to some foreign particles, however, may cause disruption of the mucus barrier. Many synthetic nanoparticles are likely to be mucoadhesive due to hydrophobic, electrostatic or hydrogen bonding interactions. We therefore sought to determine whether mucoadhesive particles (MAP) could alter the mucus microstructure, thereby allowing other foreign particles to more easily penetrate mucus. We engineered muco-inert probe particles 1 µm in diameter, whose diffusion in mucus is limited only by steric obstruction from the mucus mesh, and used them to measure possible MAP-induced changes to the microstructure of fresh human cervicovaginal mucus. We found that a 0.24% w/v concentration of 200 nm MAP in mucus induced a ∼10-fold increase in the average effective diffusivity of the probe particles, and a 2- to 3-fold increase in the fraction capable of penetrating physiologically thick mucus layers. The same concentration of muco-inert particles, and a low concentration (0.0006% w/v) of MAP, had no detectable effect on probe particle penetration rates. Using an obstruction-scaling model, we determined that the higher MAP dose increased the average mesh spacing (“pore” size) of mucus from 380 nm to 470 nm. The bulk viscoelasticity of mucus was unaffected by MAP exposure, suggesting MAP may not directly impair mucus clearance or its function as a lubricant, both of which depend critically on the bulk rheological properties of mucus. Our findings suggest mucoadhesive nanoparticles can substantially alter the microstructure of mucus, highlighting the potential of mucoadhesive environmental or engineered nanoparticles to disrupt mucus barriers and cause greater exposure to foreign particles, including pathogens and other potentially toxic nanomaterials.

## Introduction

Engineered or synthetic nanoparticles are increasingly used in diverse applications, ranging from ultra-light high strength materials to electronics, cosmetics and medicine. As the manufacturing and use of synthetic nanoparticles become more common, the potential for unintended human exposure also increases. Concerns over the safety of nanoparticles have centered primarily on their small size and, therefore, potential for deeper tissue penetration and higher reactivity (due to their high surface area to volume ratio) [1]–[3]. Recent studies have shown that some non-degradable environmental or engineered nanoparticles may trigger significant pulmonary inflammation and/or immunological responses *in vivo* [1]–[3]. Several investigators have also suggested certain synthetic nanoparticles can be directly toxic to cells through the generation of reactive oxygen species [2]–[3].

**Figure 1:**
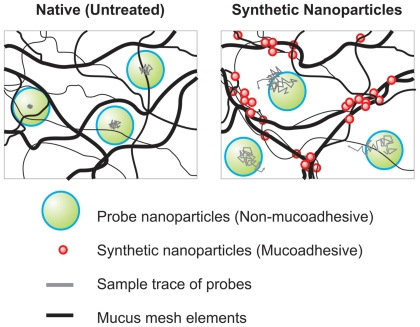
Schematic illustrating the potential effects of synthetic mucoadhesive particles (MAP) on the microstructure of mucus. MAP may increase mucus pore sizes by bundling mucin fibers through polyvalent adhesive interactions. This would allow larger non-mucoadhesive particles to penetrate mucus more readily.

Exposure to synthetic nanoparticles may occur *via* percutaneous or trans-mucosal absorption. Nevertheless, the interactions between nanoparticles and mucus in the airways, as well as other mucosal organs, have yet to be fully characterized. Mucus is a viscoelastic and adhesive gel that coats and protects nearly all exposed surfaces of the human body not covered by skin, serving as the first line of defense against foreign particles that impinge on these surfaces. The dense mucin mesh network allows mucus to efficiently trap most foreign particles through steric obstruction and/or adhesion *via* hydrophobic, electrostatic or hydrogen bonding interactions [4]. Trapped particles are quickly eliminated in as little as seconds to minutes (surface of the eye) to minutes to hours (gastrointestinal, respiratory and female reproductive tracts) by normal mucus clearance mechanisms [4]–[6]. Synthetic nanoparticles typically feature hydrophobic, charged and/or hydrogen bonding surfaces [7]–[10] and are, therefore, likely to be strongly mucoadhesive due to interactions with periodic exposed hydrophobic domains or negatively charged glycosylated segments along mucin fibers [4]. Although these and other mucoadhesive particles are unlikely to perturb the microstructure of mucus at low exposure levels, at high concentrations, they may crosslink mucin fibers and cause them to bundle together, enlarging pores in the mucus gel (Figure 1). Larger pores may compromise the ability of mucus to trap foreign particles, and increase the likelihood that other foreign particles or pathogens will reach and potentially injure the underlying epithelia.

Here, we sought to investigate the ability of synthetic mucoadhesive particles (MAP) to alter the microstructure of human mucus, using 200 nm amine-modified polystyrene nanoparticles as model MAP. We performed our studies with freshly obtained and minimally altered *ex vivo* human cervicovaginal mucus (CVM), a physiological, highly viscoelastic gel that is difficult to reproduce in cell culture. To detect changes in the mucus microstructure, we use 1 µm muco-inert particles as probes whose diffusional speeds can be related to effective pore sizes in the mucus gel. This method involves minimal perturbation of the mucus sample, in contrast to electron microscopy, which is known to introduce artifacts due to dehydration and fixation steps during sample preparation [11]–[13]. We have recently employed this technique to characterize the pore size of native CVM [14] and cystic fibrosis sputum [15]. The speeds of the probe particles also allow us to estimate the fraction of foreign particles that may penetrate a physiologically thick mucus layer over time, providing another assessment of how MAP may compromise the mucus barrier.

## Materials and Methods

### Ethics statement

Research ethics approval for the collection of human CVM samples used in this study was received from the Institutional Review Board of the Johns Hopkins University. Written informed consent was obtained from each participant enrolled in the study.

### Human cervicovaginal mucus collection

Mucus samples were obtained from women of reproductive age (ranging from 18 to 27 years old) with healthy vaginal micro-flora. Donors stated they had not used vaginal products nor participated in unprotected intercourse within 3 days prior to donating. Undiluted CVM, averaging 0.3 g per sample, was collected using a self-sampling menstrual collection device following protocols approved by the Institutional Review Board of the Johns Hopkins University [8], [16]. The device was inserted into the vagina for approximately 30 s, removed, and placed into a 50 mL centrifuge tube. Samples were centrifuged at 1000 rpm for 30 s to collect the secretions. Collected mucus was stored at 4 °C until used for microscopy the same day. The samples were collected at random times throughout the menstrual cycle, but none were obtained during ovulation based on the absence of spinnbarkeit by visual inspection. Samples that were non-uniform in color or consistency were discarded.

### Nanoparticle preparation and characterization

For model MAP, we used amine-modified polystyrene nanoparticles sized 200 nm (Molecular Probes, Eugene, OR), which were concentrated to 8% w/v (∼10^13^ particles/mL) by centrifugation and resuspension in water. These particles feature a hydrophobic core and a positively charged surface at the native pH of CVM (pH∼4), and may adhere to mucus through hydrophobic interactions with oily domains along mucin fibers or through electrostatic and/or hydrogen bonding interactions with the negatively charged, glycosylated domains of mucins [4]. For muco-inert probe particles, we covalently modified fluorescent, carboxylated polystyrene particles sized 1 µm (Molecular Probes, Eugene, OR) with 2 kDa amine-modified poly(ethylene glycol) (PEG; Nektar Therapeutics, San Carlos, CA) *via* a carboxyl-amine reaction, as published previously [8]. PEG is a hydrophilic and uncharged polymer that, at high surface density and low MW, can effectively shield the hydrophobic polystyrene core from adhesive interactions with mucins, while also minimizing interpenetrating network effects (polymer chain entanglements) and hydrogen bonding between PEG chains and mucins [8], [17].

Particle size and ξ-potential (Table 1) were determined by dynamic light scattering and laser Doppler anemometry, respectively, using a Zetasizer Nano ZS90 (Malvern Instruments, Southborough, MA). Size measurements were performed at 25°C at a scattering angle of 90°. Samples were diluted in 10 mM NaCl solution (pH 4 or pH 7) and measurements performed according to instrument instructions. A near-neutral ξ-potential at pH 7 was used to confirm PEG conjugation onto the 1 µm probe particles [8]. Based on our previous findings, muco-inert particles 1 µm or larger in diameter are highly hindered in native human mucus by steric obstruction, whereas 500 nm muco-inert particles are nearly freely diffusive due to the availability of sufficiently large spaces or pores in the mucus mesh [14], [18]. As such, 1 µm muco-inert particles are sensitive probes for detecting increases in mucus pore size.

**Table 1:** Characterization of mucoadhesive (MAP) and muco-inert (PEG-coated) probe particles.

Size, nm^a^	Surface Chemistry	Diameter, nm	ζ-potential, mV	
			pH 4	pH 7
200	NH_2_	245±1.0	13±1.1	0.1±0.1
1000	PEG	1050±7.5	−1.6±0.9	−6.1±0.9

a Provided by the manufacturer.

### Multiple particle tracking

Each mucus sample was separated into aliquots and treated with 8% w/v MAP or, as controls, (i) saline, (ii) a diluted solution of MAP (0.02% w/v) or (iii) 8% w/v of 200 nm muco-inert particles (prepared and characterized as described above for 1 µm probe particles). Solutions were added to mucus at 3% v/v, with gentle stirring to achieve visually uniform particle distribution, yielding final particle concentrations of 0.24% w/v or 0.0006% w/v. The concentration of 0.24% w/v falls within dose limits (0.17–0.35% w/v) estimated based on the Occupational Safety and Health Administration's Permissible Exposure Limits for airborne particulates, such as metal oxide nanoparticles (∼10 mg/m^3^, 8 hr time-weighted average [19]); an average breathing rate of 3.2 m^3^/hr for heavily active adults [20]; a deposition fraction of ∼5–10% for 0.1–1 µm particles in the tracheobronchial (TB) region of the lungs [21]; a TB surface area of 2741 cm^2^ [22]; and an average TB mucus layer thickness of 30 µm [4]. Treated samples were incubated at least 15 min prior to addition of diluted probe particle solutions (∼10^10^ particles/mL) at 5% v/v. Samples were transferred to custom-made chambers holding 20–30 µL of mucus each and incubated 2 hr before microscopy. Samples incubated at room temperature or at 37°C yielded similar results. The trajectories of the fluorescent probe particles in CVM were recorded using a silicon-intensified target camera (VE-1000, Dage-MTI, Michigan, IN) mounted on an inverted epifluorescence microscope equipped with 100X oil-immersion objective (numerical aperture 1.3). Movies were captured with Metamorph software (Universal Imaging Corp., Downingtown, PA) at a temporal resolution of 66.7 ms for 20 s. The tracking resolution was 10 nm, as determined by tracking the displacements of particles immobilized with a strong adhesive [23]. Trajectories of n>100 probe particles were analyzed for each experiment, and six experiments were performed in independent CVM samples from different donors. The coordinates of particle centroids were transformed into time-averaged mean squared displacements (MSD), calculated as <Δ*r* ^2^(τ)> =  [*x*(*t* + τ) – *x*(*t*)]^2^ + [*y*(*t* + τ) – *y*(*t*)]^2^ (where τ  =  time scale or time lag), from which individual particle and ensemble average MSD and effective diffusivities (D_eff_  =  MSD/(4τ) for 2D particle tracking) were calculated, as previously demonstrated [8], [24]. Immobile particles are defined as those with an average MSD below the 10 nm tracking resolution at a time scale of 1 s.

### Mucus pore size analysis

The sizes of pores between mucin fibers of saline- and MAP-exposed CVM were estimated based on an obstruction-scaling model originally developed by Amsden and coworkers for covalently cross-linked hydrogels, but equally applicable to gels with physical entanglement cross-links, such as mucus [25]–[28]. The model is valid when there is no chemical interaction between probe particles and the gel mesh, and particles traveling through the pores of the gel experience the viscous drag of water. We have shown previously that particles well coated with low MW PEG exhibit minimal adhesive interactions with mucus constituents [8], [14], [17], as well as other synthetic particles (unpublished observations). The model describes the ratio of diffusion in a gel and diffusion in water as *D*_g_ */D*_o_  =  exp((−π/4)((*r*_s_ + *r*_f_)/(*r*_g_ + *r*_f_))^2^), where *D*_g_ is the diffusion coefficient of the probe particle in the polymer gel, *D*_o_ is its diffusion coefficient in water, *r*_s_ is the particle radius, *r*_f_ is the gel fiber radius, and *r*_g_ is the effective radius of the pore. An *r*_f_ of 3.5 nm was used as the current best estimate for the radius of individual mucin fibers from biochemical, electron microscopy and atomic force microscopy observations [27]–[28].

### Simulation of particle penetration

To estimate particle penetration across mucus, we performed a Monte Carlo simulation of one-dimensional particle transport across a mucus slab of uniform thickness. The simulation consisted of ∼20,000 particles with effective diffusivities equal to those measured experimentally for the 1 µm PEG-coated probe particles in saline- or MAP-exposed CVM at a time scale of 1 s (30 particles simulated per measured diffusivity). Particles were initially located at one surface of the slab, and then allowed to undergo Brownian motion. Particle positions over time (in steps of 1 s) were recorded, and the fraction of penetrable particles was calculated as the fraction that reaches the opposite surface of the mucus slab.

### Bulk rheological characterization of MAP-exposed CVM

Bulk rheological characterization of CVM was performed with a strain-controlled cone and plate rheometer (ARES-100, Rheometrics, Piscataway, NJ). CVM from 4–5 donor samples was pooled (total volume ∼1.3 mL) and stored at 4°C until use. The temperature of specimens was maintained at 37°C during measurements. Oscillatory deformations of small amplitude (1% strain) and controlled frequency were applied to extract the frequency-dependent viscoelastic properties with minimal shearing damage to the CVM samples. We report the frequency-dependent elastic and viscous moduli, G′(ω) and G″(ω), which are the in-phase and out-of-phase components, respectively, of the stress induced in the CVM samples divided by the maximum amplitude of the applied deformation. Phase angle is defined as arctan(G″/G′); a phase angle of 90° corresponds to a viscous liquid, while 0° corresponds to an elastic solid. The rheology of control and MAP-exposed CVM was evaluated in sequential order. As with particle tracking experiments, MAP (8% w/v) were added at 3% v/v, gently stirred to ensure exposure of the entire sample, and incubated at least 15 min prior to subsequent measurements.

### Statistical analysis

All data are presented as a mean with standard error of the mean (mean ± SEM) indicated. Statistical significance between saline and MAP conditions was determined by a one-tailed, non-parametric Wilcoxon signed-rank test, since the response of mucus samples to MAP depends on a number of factors (see Discussion) and cannot be assumed to be normally distributed. *p* values less than 0.05 were considered statistically significant.

## Results and Discussion

### Effect of MAP on mucus microstructure

**Figure 2:**
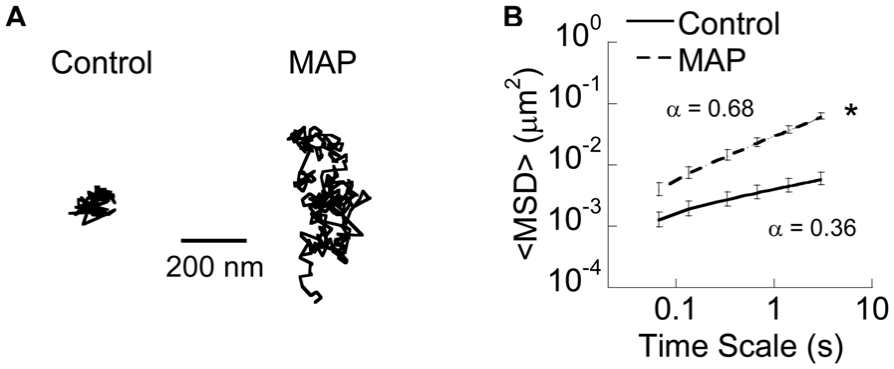
Transport of probe particles in saline- (Control) or MAP-exposed CVM. (*A*) Sample trajectories of probe particles with effective diffusivities within one S.E.M. of the mean at a time scale of 1 s. (*B*) Ensemble-averaged geometric mean square displacements (<MSD>) as a function of time scale for probe particles. Data represent six independent experiments with samples from different donors (n≥100 particles per experiment). α represents the slope of the log-log <MSD> vs. time scale plot. * indicates statistical significance at *p*<0.05.

**Figure 3:**
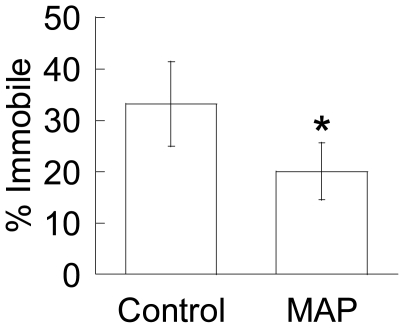
Fraction of immobile probe particles in saline- (Control) or MAP-exposed CVM. * indicates statistical significance at *p*<0.05.

In agreement with our previous work [14], [18], the diffusion of 1 µm muco-inert (PEG-coated) probe particles was strongly hindered in control mucus samples treated with 3% v/v saline, as evident by their constrained and non-Brownian trajectories (Figure 2A and Video S1). In contrast, the probes exhibited more diffusive trajectories in aliquots of the same native mucus samples treated with 3% v/v MAP at a toxicologically relevant dose of 0.24% w/v final concentration (see Methods for details; Video S2). As we have previously observed [8], MAP were nearly completely immobilized in mucus (data not shown), whereas similarly sized and larger (up to at least 500 nm) muco-inert particles diffuse freely through mucus, suggesting MAP must be immobilized by adhesive interactions to the mucin mesh network. Using multiple particle tracking [8], [24], we quantified probe particle motions in terms of ensemble average mean square displacements (<MSD>; Figure 2B). Five out of six independent samples showed an increase in probe particle <MSD> upon exposure of mucus to MAP, while one showed no significant change. On average, MAP exposure increased probe particle effective diffusivity by ∼10-fold for each sample, at a time scale of 1 s. This improvement was also reflected by the slope α of the log-log <MSD> vs. time scale plot (α = 1 represents unconstrained Brownian transport, while decreasing α corresponds to increased obstruction to particle movement). The average α was 0.68 for probe particles in treated samples, compared to only 0.36 in control samples. The increase in particle transport rates was due in part to a significant drop in the fraction of immobile probes (from 33% to 20%; Figure 3).

Since well-PEGylated particles have minimal affinity to mucin fibers [8], [17], the increase in probe particle speeds in MAP-exposed mucus suggests MAP enlarged the pores in the mucus mesh. To quantify this change in the mucus microstructure, we fitted the effective diffusivities of the probe particles to an obstruction-scaling model, which relates the obstruction experienced by non-interacting solute particles to the pore size of the surrounding mesh network [25]–[26]. The average pore size of mucus samples increased significantly from 380±30 nm in the saline control to 470±80 nm in the MAP-exposed condition (*p*<0.05). The fraction of pores with spacings ≥500 nm increased from 11% to 19%, and the fraction of pores ≥1 µm increased from 3% to 9%.

In contrast to our observations with a relatively high MAP dose (0.24% w/v), the same amount of 200 nm muco-inert nanoparticles (densely coated with PEG) or 400-fold less MAP did not significantly affect the mucus microstructure (data not shown), suggesting the effects of synthetic nanoparticles on mucus depend on both particle concentration and surface chemistry. Synthetic nanoparticles may adhere to mucins through hydrophobic or electrostatic interactions with hydrophobic and glycosylated domains along mucin fibers, respectively. At low concentrations, the number of MAP is likely insufficient to form the bonds with mucins (*i.e.*, avidity) necessary to crosslink or bundle mucins, which are normally held apart by entanglements or terminal disulfide bridges. Likewise, particles that interact minimally with mucins would have little effect on the mucus microstructure, even at high concentrations. However, the collective binding interactions of large numbers of MAP with multiple mucin fibers may generate sufficient avidity to cause mucins to crosslink and bundle together, thus yielding larger pores in the mucus mesh. This notion is consistent with visual observations by Olmsted and coworkers of high concentrations of fluorescent polystyrene nanoparticles agglomerating mid-cycle cervical mucins into thick cables [27]. In that work, polystyrene nanoparticles caused a striking collapse of the mucus gel, to an extent greater than what we observed here. A key difference that may explain this discrepancy is the use of ovulatory cervical mucus (OCM) by Olmsted *et al.* compared to non-ovulatory cervicovaginal mucus here. OCM is more dilute [29], so there are fewer entanglements and crosslinks to resist the displacement of mucin fibers during bundling by MAP. In addition, electron microscopy studies have suggested ovulatory mucus contains long strands of mucin fibers arranged in parallel with minimal entanglements, which may promote bundling by MAP compared to non-ovulatory mucus that consists of a random mesh of highly entangled mucin fibers [30]. Other experimental differences include the volume of particles added (20% v/v by Olmsted vs. 3% v/v here), the total amount of particles added (final concentration 2% w/v vs. 0.24% w/v here) and the degree of particle mixing into mucus (relatively rigorous vs. gentle stirring here).

We observed substantial heterogeneity in MAP-induced changes to the microstructure, which may be attributed to compositional differences between CVM samples collected at random points throughout the menstrual cycle (excluding the ovulatory phase) and from different donors. Due to hormonal changes, the contents of mucins (both total mucin concentration and different MUC types) [29], [31], lipids [32] and other mucus constituents differ markedly throughout the menstrual cycle. As discussed above, mucin concentration may determine the extent of mucin bundling possible. Lipids, which naturally coat the hydrophobic domains of mucins [33], may also interfere with adhesive interactions between mucins and MAP. It is likely that the effect of a higher particle concentration in improving the mucus-altering ability of MAP may eventually saturate, since the number of adhesive interactions mucins can form with particles is finite. In addition, physical entanglements and disulfide crosslinks within the mucin mesh network may restrict the extent of mucin bundling possible.

### Predicted particle penetration into MAP-exposed mucus layers

**Figure 4:**
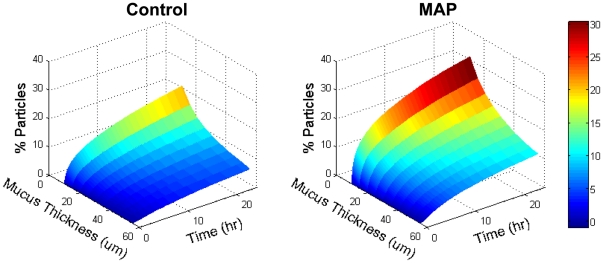
Monte Carlo simulation of particle penetration across a layer of saline- (Control) or MAP-exposed CVM over time. The simulation consists of ∼20,000 particles undergoing random diffusion with diffusivities equal to the experimentally measured diffusivities of probe particles in either condition. The % of particles able to reach the opposite side of the mucus layer is reported.

The increased mobility of the probe particles in MAP-exposed mucus suggests a greater fraction may penetrate physiologically thick mucus layers. To model the long range diffusion of the particles, we performed a Monte Carlo simulation of particles undergoing Brownian diffusion across a mucus slab, using the diffusivities of hundreds of probe particles measured above (Figure 4). The thickness of physiological mucus layers varies from tens to several hundred microns depending on anatomical location [4], [34]–[36] here, we assumed thicknesses ranging from 10 to 55 µm, which correspond to values reported for the large airways [36]. For a thickness of 10 µm, about 2-fold more of 1 µm probe particles were predicted to penetrate the mucus layer within 1 hr in MAP-exposed vs. saline-treated mucus (11% vs. 6%, respectively), and, for a thickness of 30 µm, 3-fold more probe particles were predicted to penetrate (3% vs. 1%, respectively). For comparison, if the particles were diffusing through pure water, 64% and 17% of probe particles would theoretically be able to penetrate 10 µm and 30 µm, respectively, over the same duration. The increase in probe particle penetration suggests exposure of mucus to MAP may similarly enhance penetration by pathogens or other foreign particles with muco-inert surfaces (e.g., Norwalk virus and human papilloma virus [27]), which may significantly increase risk of infection or toxicity. Nevertheless, the window of opportunity for harmful particles to penetrate the mucus barrier is likely limited, since MAP-exposed mucus is likely to be cleared by natural mucus clearance mechanisms as the mucus blanket is continuously renewed, on the order of minutes to hours depending on mucosal site [4].

### Bulk rheology of MAP-exposed mucus

**Figure 5:**
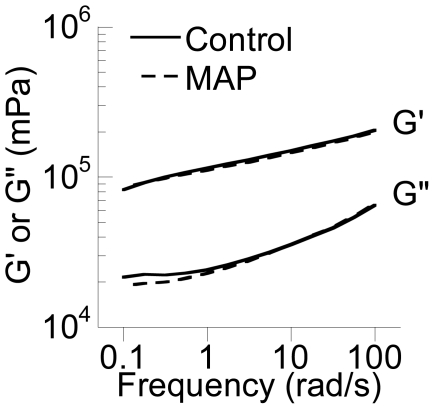
Bulk elastic (G′) and viscous (G″) moduli of unexposed (Control) and MAP-exposed pooled CVM.

Rapid mucus clearance is critical to the effectiveness of the mucus barrier. Since proper mucus clearance depends strongly on the bulk rheological properties of the mucus gel [37], we also investigated whether MAP may alter the bulk viscoelasticity of mucus. We pooled mucus from several donors and performed strain-controlled cone and plate rheometric measurements on native and MAP-exposed samples sequentially. The bulk viscous and elastic moduli were quantified by applying a small, fixed-amplitude oscillatory stress at specified frequencies. Both the viscous and elastic moduli changed minimally upon MAP exposure (Figure 5), decreasing on average by ∼4% and 2%, respectively. The phase angle (δ) for both native and MAP-exposed samples was ∼14°, indicating that mucus remained a viscoelastic solid (δ = 0° indicates a Hookean solid, δ = 90° indicates a purely viscous fluid and 0°<δ<90° indicates a viscoelastic material). These values are comparable to those we have measured before for fresh mucus [18]. Thus, although MAP may alter mucus microstructure substantially, the bulk rheology of mucus appeared unperturbed.

Classical polymer physics models [18], [38]–[40] would predict a 35–65% decrease in the bulk elastic modulus as pore size increases from 380 nm to 470 nm, in contrast to the ∼2% decrease observed here. However, the essentially unchanged bulk rheology of mucus upon MAP exposure is in agreement with our previous finding that the microstructure of mucus can be selectively altered without perturbing bulk rheology [18]. In that work, we showed that treatment of mucus with a non-ionic detergent disrupted adhesive interactions between mucins, reducing natural mucin bundling and, hence, pore sizes. We postulated that the resulting increase in the number of entanglements between mucins (expected to increase bulk viscoelasticity) likely offset the decrease in adhesive interactions (expected to decrease bulk viscoelasticity), resulting in no detectable change in the bulk rheology. Similarly, an increase in mucin fiber bundling upon MAP exposure here may result in a decrease in the number of entanglements under shear, as well as a simultaneous increase in adhesive interactions between mucins. These two opposing effects may balance each other to the extent that no change in bulk rheology was detected even with a sensitive strain-controlled rheometer. Based on visual observation of the fluorescently-labeled particles, MAP appeared to mix uniformly into the entire mucus sample; thus, it is unlikely that non-uniformly distributed MAP caused only local changes to mucus microstructure that were not detectable by bulk rheological measurements. Our results suggest proper mucus clearance, which depends critically on the bulk rheological properties of mucus, may not be directly impaired by exposure to MAP, even at relatively high exposure levels. However, it should be noted that toxic nanoparticles that bypass the mucus barrier may cause toxicity to underlying epithelial cells, which may lead to mucus hypersecretion, altered mucus bulk rheology and poor mucus clearance, similar to the pathogenesis of inflammatory lung diseases [41]–[44].

### Implications for drug delivery applications

Finally, our results may offer a unique approach for potentially enhancing drug or nucleic acid delivery to mucosal surfaces. Large drug carriers are preferred for improved drug loading and release kinetics, but even those engineered to have muco-inert surfaces are markedly slowed by steric obstruction from the mucus mesh, particularly in thicker secretions like cystic fibrosis sputum [15]. The use of biodegradable and biocompatible MAP that transiently enlarge mucus pores may significantly enhance the penetration of large drug carriers through mucus. These ‘sacrificial’ mucoadhesive nanoparticles may be employed prior to administration of the drug carriers. As mucus is continuously secreted, MAP will be displaced over time; however, mucus clearance in the vagina and other mucosal surfaces occurs on the order of several hours [4], providing sufficient time for drug carriers to penetrate MAP-altered mucus. In pilot studies, exposure of mucus to a high concentration of MAP did not cause existing MAP that were already immobilized to become mobile. This suggests that, in the absence of exposure to exogenous pathogens, the use of ‘sacrificial’ nanoparticles would not increase the risk of infection by already-present pathogens while facilitating sustained and/or targeted drug delivery to mucosal surfaces.

In practice, the amount of mixing of MAP into mucus that occurs during particle administration may be limited, depending on the mucosal surface of interest. For example, little to no mixing is expected in the respiratory tract, where aerosolized MAP would deposit and remain trapped on the surface of the mucus layer. In contrast, some mixing may occur in the cervicovaginal tract, where intra-abdominal pressure generates squeezing flows that can cause mixing of MAP into the mucus layer. Nevertheless, even if MAP are distributed only in the outermost, luminal mucus layer, they may still provide therapeutic benefits by sufficiently enlarging pores to enhance the penetration of drug carriers into deeper mucus layers. This is of particular significance at mucosal surfaces like the vagina, where secreted mucins form two distinct layers, a quickly cleared luminal mucus layer and a more slowly cleared adherent mucus layer adjacent to the epithelium. By reaching the slowly cleared adherent mucus layer, drug carriers may be retained at the mucosal surface in close proximity to target cells for prolonged periods of time, leading to enhanced efficacy.

In summary, we have characterized the effect of synthetic particles on the microstructure of native mucus gel, and demonstrated that both particle concentration and surface chemistry play a role in whether the particles disrupt the mucus barrier. High concentrations of MAP can increase pore sizes in mucus through the crosslinking or bundling of mucin fibers, allowing greater penetration of foreign particles across mucus. While we used CVM here, lung and other human mucus secretions have generally similar bulk rheological properties and compositions as CVM, with 2–5% w/w mucins (predominantly of the MUC5B mucin type [45]) and 90–98% w/w water [4], [33]. Thus, our findings may also extend to these other types of human mucus. These results highlight the importance of understanding nanoparticle-mucus interactions and their implications for nanotoxicology, as well as for biomedical applications.

## Supporting Information

Video S1 Transport of 1 µm muco-inert (PEG-coated) probe particles in human cervicovaginal mucus samples treated with 3% v/v saline, over the course of 20 s. The trajectories of the probe particles are strongly hindered.
(AVI)

Video S2 Transport of 1 µm muco-inert (PEG-coated) probe particles in human cervicovaginal mucus samples treated with 3% v/v mucoadhesive particles (MAP; 0.24% w/v final concentration), over the course of 20 s. The trajectories of the probe particles are much more diffusive than in the same mucus samples treated with saline.
(AVI)
